# CFTR Modulator Therapies: Potential Impact on Airway Infections in Cystic Fibrosis

**DOI:** 10.3390/cells11071243

**Published:** 2022-04-06

**Authors:** Francesca Saluzzo, Luca Riberi, Barbara Messore, Nicola Ivan Loré, Irene Esposito, Elisabetta Bignamini, Virginia De Rose

**Affiliations:** 1 Emerging Bacterial Pathogens Unit, Division of Immunology, Transplantation and Infectious Diseases, IRCCS San Raffaele Scientific Institute, 20132 Milan, Italy; saluzzo.francesca@hsr.it; 2 Postgraduate School in Respiratory Medicine, University of Torino, 10124 Torino, Italy; luca.riberi@unito.it; 3 Adult Cystic Fibrosis Centre, Azienda Ospedaliero-Universitaria San Luigi Gonzaga, 10043 Orbassano, Italy; barbara.messore@gmail.com; 4 WHO Collaborating Centre and TB Supranational Reference Laboratory, Emerging Bacterial Pathogens Unit, IRCCS Ospedale San Raffaele, 20132 Milan, Italy; lore.nicolaivan@hsr.it; 5 Paediatric Pulmonology Unit, Regina Margherita Hospital AOU Città della Salute e della Scienza, 10126 Torino, Italy; espirene75@gmail.com (I.E.); elisabetta.bignamini@gmail.com (E.B.); 6 Department of Molecular Biotechnology and Health Sciences, University of Torino, 10126 Torino, Italy

**Keywords:** cystic fibrosis, CFTR, modulators, infections, airway microbiology, pathogens, therapies

## Abstract

Cystic Fibrosis (CF) is an autosomal recessive disease caused by mutations in the gene encoding for the Cystic Fibrosis Transmembrane conductance Regulator (CFTR) protein, expressed on the apical surface of epithelial cells. CFTR absence/dysfunction results in ion imbalance and airway surface dehydration that severely compromise the CF airway microenvironment, increasing infection susceptibility. Recently, novel therapies aimed at correcting the basic CFTR defect have become available, leading to substantial clinical improvement of CF patients. The restoration or increase of CFTR function affects the airway microenvironment, improving local defence mechanisms. CFTR modulator drugs might therefore affect the development of chronic airway infections and/or improve the status of existing infections in CF. Thus far, however, the full extent of these effects of CFTR-modulators, especially in the long-term remains still unknown. This review aims to provide an overview of current evidence on the potential impact of CFTR modulators on airway infections in CF. Their role in affecting CF microbiology, the susceptibility to infections as well as the potential efficacy of their use in preventing/decreasing the development of chronic lung infections and the recurrent acute exacerbations in CF will be critically analysed.

## 1. Introduction

Cystic Fibrosis (CF) is an autosomal recessive disease caused by mutations in the gene encoding for the Cystic Fibrosis Transmembrane Conductance Regulator (CFTR) protein. CFTR is a cAMP-activated chloride channel which regulates ion transport across the apical membrane of epithelial cells; its dysfunction causes ion imbalance, depletion of the airway surface fluid and pH alteration which lead to an impairment of mucociliary clearance and host immune defences and ultimately to a higher susceptibility to chronic airway infections [1,2,3,4].

Although CF is a complex multiorgan disease, lung disease, characterized by chronic inflammation, progressive airflow obstruction, and airway bacterial infections, represents the major cause of morbidity and mortality in patients with this disease [1,2,3,4]. The mechanisms linking CFTR genetic dysfunction to chronic airway infection and inflammation are not yet fully defined. As outlined above, CFTR dysfunction induces an alteration of the airway microenvironment with airway surface dehydration, mucus hypersecretion and accumulation in the airway lumen and impaired mucociliary clearance. This favours the development of chronic airway infection which in turn leads to the activation of an exuberant and persistent inflammatory response; a vicious cycle thus occurs between infection and inflammation, leading to progressive and irreversible lung damage and ultimately to respiratory failure [5,6,7,8,9,10,11,12].

Recently, novel therapies aimed at correcting the basic CFTR defect have become available (Table 1) and have led to substantial clinical improvement of CF patients, altering disease progression. During the last few years, progress in the development of CFTR modulators has advanced in application from a small CF population with a specific and rare genotype, to a broad population that includes those with one or two copies of the most common F508del CFTR mutation, thus addressing up to 90% of individuals with CF [13,14,15,16].

There is now abundant evidence that CFTR modulators can restore or increase the biologic function of CFTR, although the bioelectric and clinical effects of these medications vary by genotype. Restoration of CFTR function has been associated with improved airway surface hydration and mucociliary clearance in addition to increased airway surface fluid pH. All these changes in the pulmonary microenvironment can improve endogenous defence mechanisms and influence microbiology and bacterial infections in the CF airways. CFTR modulators might thus affect the development of chronic airway infections and/or improve the status of existing infections in CF patients. However, the full extent of the effects of CFTR-modulator therapy, especially in the long-term, is still unknown. Some of the major clinical effects are: an important reduction in the number of exacerbations, increase of pulmonary function (FEV1) and body weight, as well as the reduction of sweat chloride concentration.

Several studies are ongoing addressing the crucial role of novel modulator therapies in decreasing the long-term incidence/prevalence of chronic and recurrent airway infections, and it has been observed that certain CFTR modulators are capable of decreasing the prevalence of bacteria, particularly the key CF pathogen *Pseudomonas aeruginosa* (*P. aeruginosa*) in treated patients. Furthermore, some in vitro studies show a synergistic interaction between CFTR modulators and certain antibiotics. Nonetheless, even considering these encouraging results, contrasting findings have been reported.

Hence, it remains challenging to fully understand the results of these studies, particularly in light of the recent changes in CF infections epidemiology [16].

This review aims at providing an overview of current evidence on the potential impact of CFTR modulators on airway infections in CF. Their role in affecting CF microbiology and the susceptibility to infections as well as the potential efficacy of their use in preventing/decreasing the development of chronic lung infections and the recurrent acute exacerbations in CF will be critically analysed.

## 2. Airway Infections in Cystic Fibrosis

Patients with CF are susceptible to chronic airway infections and recurrent infectious exacerbations that are the leading cause of morbidity and mortality for these patients [1,2,3]. CF airway pathogens play a critical role in the progression of lung disease [17]; the persistence of infection, in fact, and the recurrent infectious exacerbations mostly contribute to the chronic and exaggerated inflammatory response in the CF airways, leading to tissue damage and the subsequent lung function impairment [5,9,11].

CF airways represent a heterogeneous environment in continuous evolution, whose characteristics may affect the interactions between the host immune system, microbiota, and pathogen(s) [18,19]. These characteristics of the CF airway environment complicate our understanding of the establishment and progression of infection [20]. On the other hand, a better definition of mechanisms of airway infection as well as of the dynamics of microbiome changes with disease progression and in response to treatment plays an essential role in developing novel and more effective strategies for managing airway infections [18].

The use of aggressive antibiotic therapy and airway clearance, as well as the implementation of infection control guidelines had a key role in CF infection control; however, airway infection prevention and treatment continue to represent a relevant challenge in both children and adults with CF [21]. Whether and how the recent introduction in clinical practice of CFTR modulator therapy will affect the development and progression of airway infections in CF patients is still at the beginning of being deciphered.

The most common CF airways pathogens are *Staphylococcus aureus* (SA), *Haemophilus influenzae* (HI), *P. aeruginosa*, and *Burkholderia cepacia complex* (BCC) as well as *Stenotrophomonas maltophilia* (SM), and *Achromobacter xylosoxidans* (AX) which now have an emerging role in CF infections [22]. Recently, the occurrence of MDR *P. aeruginosa*, methicillin-resistant *S. aureus* (MRSA) and non-tuberculous mycobacteria (NTM) is raising considerable concern [23].

SA has been reported as one of the first pathogens isolated in CF airways, detectable in more than 50% of children aged ≤2 years old and in almost 80% of teenagers [23,24]. While Methicillin-Susceptible SA (MSSA) shows a higher prevalence in the age group below 15 years old, MRSA occurrence increases between 10 and 30 years old [23]. In several CF patients SA detection persists over time [25,26]; in these cases, usually a predominant clone tends to adapt colonizing or chronically infecting the CF lung environment [25,27] using several different mechanisms from genome rearrangements [28] to the emergence of small colony variants (SCVs) [29]. The effects of the persistence of SA on the clinical outcome of CF patients are still not clearly defined, as it is still difficult to determine the difference between colonization and infection [30]. Moreover, while the association between *P. aeruginosa* chronic infection and the progression of the lung disease is well established [31], SA persistence has been associated to a worsening pulmonary function only when associated to the detection of SCVs [32,33].

Chronic airway infection by *P. aeruginosa* has been associated to a relentless and rapid decline of CF patients’ lung function as well as to the development of MDR infections and poor response to antibiotic therapy [34,35].

The advent of next-generation sequencing (NGS) and the increased interest in the study of the microbial communities and microbiome had led to the knowledge that the co-existence of other microorganisms can influence *P. aeruginosa* infection characteristics and promote or hamper the infection chronicization [35,36]. Moreover, CF polymicrobial communities can influence antimicrobial resistance, expression of virulence factors and response to antimicrobial therapy, potentially leading to a poorer clinical outcome [18,21,35,36,37].

Genotypic and phenotypic characteristics of CF pathogens might be modulated by the CF airway environment; this has been shown for *P. aeruginosa*, but is likely to occur for other CF pathogens. Modulator drugs that induce beneficial changes in the airway environment might thus affect gene expression of CF pathogens and induce changes in pathogen behavior; therefore, it is possible that modulators effects on CF pathogens might contribute to changes in CF airway microbiology in the future; however, to date these effects remain insufficiently investigated. Intriguingly, some studies also suggested that some CF pathogens may affect the modulators effect either by increasing or by decreasing it; however, these results are still controversial, but certainly deserve further evaluation [38,39,40,41].

In addition to the effect of the airway microenvironment and of polymicrobial community interactions, host–pathogen interactions and the airway inflammatory/immune responses also affect the development/persistence of infection as well as disease progression in CF.

Immune response to CF pathogens has been dissected and characterized for the most common and relevant pathogens, such as SA [42,43], *P. aeruginosa* [44,45,46] and HI [47,48,49]. 

As we already stated, among CF pathogens, *P. aeruginosa* plays a critical role in promoting chronic infection and structural alterations in the CF lungs [50]. Chronic infection by this microorganism fuels a persistent and exaggerated inflammatory response and is associated with reduced lung function and poor prognosis [9,50]. Chronic infection is induced by *P. aeruginosa* variants characterized by adaptive traits and different pathogenicity. Both innate and adaptive host defences play an important role in modulating the immune response to this microorganism. In particular, in the context of innate immunity, the activation of inflammasome by NOD-like receptors plays a pivotal role in modulating host response to *P. aeruginosa* [51]. Interestingly, studies in both children and adults with CF suggest a modulation of Tregs by chronic *P. aeruginosa* infection of the lung. Hector and colleagues found a negative correlation between Tregs levels in blood and bronchoalveolar lavage fluid, and Pseudomonas infection in children with CF [52]. The same group treated human peripheral blood mononuclear cells (PBMCs) with cell-free supernatants from different *P. aeruginosa* strains and showed that particularly virulent and flagellin-deficient strains seem to decrease Tregs in CF [52,53]. In adult CF patients, chronic *P. aeruginosa* lung infection was associated with impaired proportion of peripheral Tregs.

Similarly to *P. aeruginosa* infections, dysregulated inflammatory responses to SA have been observed in animal models [54]. The appearance of SCVs during SA infection may represent a mode of growth to promote chronic persistence. Wieneke et al. showed that SA airway persistence in CF is characterized by a highly diverse and dynamic microbial population and associated with *P. aeruginosa* coinfection and inflammation [55].

Chronic infection and an exaggerated inflammatory response are key drivers of CF pathophysiology and particularly of CF lung disease. Disruption of microbiota homeostasis triggers the immune response and favours the progression of CF lung disease; the altered environment of CF airways with impaired oxygen availability and altered pH as well as the decreased mucociliary clearance allow airway colonization by pathogenic species. The concurrent impairment of innate and adaptive immunity favours the occurrence of chronic infections [56]. An imbalance of pro- and anti-inflammatory mediators, including dysregulated Th2/Th17 cells and impairment of regulatory T cells, contributes to the altered immune response to classical CF pathogens and to the persistence of airway inflammation.

The beneficial effects of the modulator drugs on mucus hydration and mucociliary clearance as well as on pH or the chemical composition of airway secretions might be predicted to positively affect the airway microenvironment and thus hinder either the appearance or the persistence of pathogens at this level. On the other hand, modulators effects on airway microbiology are likely to be variable, being also affected by factors such as different response to treatment, patient age, severity of disease, and the extent of airway damage. Furthermore, in addition to indirectly affecting airway microbiology by addressing CFTR dysfunction, some CFTR modulators might have direct antimicrobial properties [37].

Some studies have suggested that CFTR modulators may also decrease the inflammatory response in CF airways, an effect that would further contribute to improved disease outcomes [57]; however, to date conflicting results have been reported and data still remain inconclusive (reviewed in [58,59]). Furthermore, it remains to be defined whether these drugs are capable of restoring dysfunctional CFTR in immune cells that may contribute to the exaggerated inflammatory process and the impaired clearance of pathogens in CF airways [60,61].

Interestingly, in this context, Zhang et al. [62] reported that Ivacaftor (IVA) was capable of improving phagocytosis of human CF monocyte-derived macrophages, an effect that was associated with a modest improvement of bacterial killing; IVA also induced a decrease of inflammatory cytokine production by macrophages. Very recently, Hisert et al. [63] studied a small group of adult CF patients with chronic airway infections before and 7 days after initiation of IVA; in these patients, by using unbiased “omics” methods, they assessed the changes in inflammatory phenotypes of blood monocytes induced by IVA. They showed that multiple transcriptional programs, including pathways associated with immunity and inflammation, were upregulated in circulating monocytes after IVA treatment. A significant increase of plasma levels of the myeloid chemokines CCL2 and CXCL2 and an overall improvement of FEV1 were also observed.

Further dissection of specific inflammatory profiles and immune responses associated to the persistence of CF pathogens is essential to determine the immune axis regulating the exaggerated inflammatory response, and thus to better define the strategies for counteracting tissue destruction and lung disease progression. In this context, it will also be very important to evaluate whether CFTR modulator therapy might influence innate and adaptive immune dysregulation in CF, and eventually synergize with anti-inflammatory therapy in modulating the altered inflammatory/immune response to chronic infection in CF.

## 3. CFTR Modulators and CF Microbiology

As we already emphasized, CFTR modulators are reshaping CF treatment. These drugs have led to substantial clinical improvement of CF patients, altering disease progression, and dramatically affecting quality of life of these patients. As we will discuss later, in particular the newest combination of Elexacaftor, Tezacaftor and Ivacaftor (ETI), that has proved remarkably effective in improving lung function and other clinical outcomes, could be suitable to treat almost 90% of people with CF, finally increasing patients life expectancy [15,16,64,65]. 

Although a significant number of studies have focused on physiological and clinical effects of CFTR modulators, there remains yet no clear understanding of the effects of these drugs on lung infections, a crucial manifestation of CF largely affecting the prognosis of the disease, and some inconsistent results have been reported.

The lung microbiome of patients with CF has been well characterized and several studies have reported that, with the progression of the disease, the CF lung microbiome diversity decreases, with predominance of opportunistic pathogens, such as *P. aeruginosa* [66,67,68,69,70]. Assessing the airway microbiome changes in response to CFTR modulators is therefore important for improving therapeutic strategies aimed at management of airway infections [37]. IVA, a modulator for the treatment of patients with at least one G551D allele has been the main focus of the studies concerning the effects of CFTR modulators on the airway microbiome; recent studies also investigated the effect of Lumacaftor (LUMA)-IVA, Tezacaftor (TEZA)-IVA and during the past few years a great deal of interest has emerged with the introduction of the highly effective triple combination ETI.

Nonetheless, a consensus regarding the effectiveness of new modulator treatments on CF airways infections has yet to be reached. Studies on CF lung microbiota pre- and post-IVA treatment demonstrated a shift towards a “healthier” microorganism community composition that correlated with the levels of circulating inflammatory markers [71,72]. However, the effect of this modulator on chronic infections is still to be proven as the long-term bacterial load of *P. aeruginosa* does not appear to be affected by this treatment [73,74]. This has been determined by several studies that demonstrated a rapid reduction of *P. aeruginosa* density in CF patients’ sputum immediately after the beginning of the therapy followed by a rebound at the pre-treatment levels [71,73]. Moreover, a study following up CF patients for 6 years after IVA initiation has demonstrated that the bacterial burden, assessed through cultural methods, remained stable at the pre-treatment levels for all the observation periods [75]. The addition of CFTR correctors such as LUMA has shown an increase in microbial diversity in the CF airways in some studies [76] but other studies show a less marked response [77]. Neerincx et al. [77] showed that in CF patients homozygous for the F508del CFTR mutation, treatment with LUMA/IVA for 6 months induced a temporary and moderate change in the lung microbiome mainly characterised by a reduction in the relative abundance of *P. aeruginosa*. A similar trend was observed in sputum metabolome, in particular for metabolites from the tryptophan–kynurenic acid pathway that seems to be related to the abundance of *P. aeruginosa*. Taken together, these studies do not support the concept that LUMA/IVA treatment in CF patients has important effects on microbiological composition.

A recent study analysing sputum microbiome and metabolome in a small number of CF patients (*n* = 24) before and after ETI therapy showed that this treatment was associated with changes in the airway microbiome and metabolome [78]. The drug effect was stronger on sputum biochemistry with a decrease in peptides, amino acids and kynurenine metabolism; these findings suggest that ETI therapy and possibly other CFTR modulators [77] might “reshape microbiome niche space in CF mucus” by decreasing the availability of peptides and amino acids, an effect that could have clinical implications for the treatment of lung infections. However, more long-term studies on larger number of patients are needed to define the effects of ETI treatment and other modulators on airway microbiome as well as to assess whether these treatments will induce an altered steady state of the bacterial and viral community.

Recent studies have also evaluated the potential synergistic effects of CFTR modulators and antibiotics. The effectiveness of IVA associated to antibiotics in eradicating *P. aeruginosa* and SA chronic infections in CF was recently assessed by Durfey et al. (2021) in 10 CF patients chronically infected by *P. aeruginosa*, SA or both. A reduction in bacterial burden was observed that; however, resulted transient in most of the patients as the bacterial load rebounded as soon as antibiotic therapy was stopped. The only two subjects who successfully cleared the infection had the lowest baseline sweat chloride values and reached the lowest values after IVA, suggesting that the infection clearance could be associated at least in part to the amount of CFTR activity reached after treatment [79,80].

In vitro studies by Cigana et al. [81] suggest that some CFTR modulators (LUMA, TEZA and in particular IVA) have bactericidal and bacteriostatic activity against SA and can synergize with common antibiotics against SA and *P. aeruginosa*. Recent observations by the same group extend these findings to Elexacaftor and ETI, showing that both have antimicrobial activity against SA, but not against *P. aeruginosa*. When combinations of CFTR modulator and common antibiotics were tested, isolate-dependent additive effects were observed for both SA and *P. aeruginosa*. Elexacaftor and ETI were shown to potentiate the activity of amoxicillin, teicoplanin, vancomycin and azithromycin in some SA isolates; regarding *P. aeruginosa*, ETI potentiated the antimicrobial activity of ciprofloxacin, meropenem and tobramycin in some *P. aeruginosa* isolates whereas it showed a striking additive effect with colistin or polymyxin B on most *P. aeruginosa* isolates, and the activity seems to be mainly due to IVA. These findings thus suggest that CFTR modulators can affect antimicrobial susceptibility of key CF pathogens possibly by modifying their transcriptome and genome profile. Further studies are needed to extend our knowledge of these modulators effects that might have a relevant clinical impact.

Interestingly, very recent in vitro studies evaluating the effects of ETI on CF neutrophils, an inflammatory cell playing a crucial role in CF pathophysiology, showed that the triple combination was able to increase CFTR cellular expression and potentiate the antimicrobial mechanisms of CF neutrophils [82].

### Possible Causes of Infection Persistence

Several causes have been investigated to explain the long-term recurrence of chronic *P. aeruginosa* and SA infections in CF patients treated with modulators. One of the possible explanations could be that the airway epithelial cell damage associated with the development of structural airways disease could limit the effect of CFTR modulators in specific regions finally facilitating the recurrence of the same bacterial loads [79,83].

Macrophages also play an important role in modulating the development of chronic infection as well as in infection recurrence. Recent studies have shown the CFTR modulators’ capability of improving macrophages phagocytic capacity as well as cytokine production [84]. However, the association of LUMA/IVA seems to have only a limited impact in rescuing CF macrophage function in patients with severe mutations [85].

Furthermore, as emphasized above, more severe structural airway damage might persist despite highly effective modulator therapy and favours the development of chronic airway infection. Nonetheless, case reports published in the last two years described the clearance of BCC [86] and *M. abscessus* [87] chronic infections after the beginning of the therapy with modulators.

Finally, it is important to highlight that CF airways can be colonized and chronically infected by community of microorganisms which adapt over time, favouring the persistence of pathogens presenting low metabolic activity and high tolerance to killing [88,89]. In this environment modulators can have a lower impact on the eradication of chronic infection even if CFTR-dependent host response has been corrected.

## 4. CFTR Modulators and Chronic Airway Infections/Recurrent Exacerbations

### 4.1. CFTR Modulators and Chronic Airway Infections

As we already emphasized, CFTR modulators discovery and introduction in the clinical arena had a dramatic impact on the CF patient population. However, little is known about the full extent of their effects as well as on the long-term effects in particular as far as chronic airway infections and infectious exacerbations are concerned. Up to now, in fact, there are mainly short-term studies, and fewer experiences regarding long-term effects.

As we already outlined, to date, CFTR modulator studies have focused largely on clinical outcomes, including lung function, symptoms, sweat chloride levels and disease exacerbations; the evaluation of changes in airway microbiology and the effects on development and/or persistence of chronic airway infections were not addressed in depth.

#### 4.1.1. Ivacaftor

IVA was the first CFTR modulator approved for clinical use in 2012. Extensive data in different patient populations have documented that IVA modifies the course of the disease in CF patients, slowing disease progression. One of the first studies evaluating the effects of IVA therapy on *P. aeruginosa* sputum load as well as on other clinical outcomes was that of Rowe et al. [90] who studied 133 patients aged 6 years and older treated with IVA for 6 months. In addition to significant improvements in clinical parameters (sweat chloride, lung function, BMI, and mucociliary clearance), the investigators observed a reduction in *P. aeruginosa* burden after 6 months of treatment and a downward trend in the relative abundance of traditional CF bacterial pathogens, with a significant increase in the relative abundance of *Prevotella*. Consistent with these results are the findings of the GOAL study, a longitudinal observational study of 151 US patients aged 6 years and older with at least 1 copy of the G551D mutation treated with IVA; results were linked with retrospective and prospective culture data from US CFF Patient Registry [91]. In this study the changes in CF respiratory pathogens the year before and after the treatment were assessed and correlated with the clinical response. In 29% of patients with culture positive for *P. aeruginosa* the year prior to IVA use, negative cultures were reported the year after treatment; 88% of those *P. aeruginosa* free remained uninfected [91]. Overall, IVA treatment reduced the odds of *P. aeruginosa* positivity by 35% (OR 0.65; 95% CI 0.53–0.79, *p* < 0.001) with shift in patients’ *P. aeruginosa* infection category (free, intermittent or persistent infection according to Leeds criteria): 70% of patients with intermittent *P. aeruginosa* infection were infection free after IVA treatment, compared to only 10% of those with persistent infection (*p* < 0.001). This microbiological benefit was more remarkable in patients with less severe lung disease. A similar decreasing trend was observed in the prevalence of *Aspergillus fumigatus* positivity (OR 0.47%; 95% CI 0.23–0.96) while no changes in *S. aureus* (MSSA OR 0.93; 95% CI 0.71–1.21 and MRSA OR 1.13; 95% CI 0.84–1.52) or other common CF pathogens were observed.

In a subsequent prospective study [73] in which the impact of IVA treatment was assessed in 12 adult patients with G551D mutations-8 chronically infected with *P. aeruginosa*, and 2 with *B. Cepacia*—the treatment with the potentiator decreased sputum *P. aeruginosa* load and markers of inflammation during the first year of treatment. However, in none of the subjects was *P. aeruginosa* eradicated and after the first year of treatment *P. aeruginosa* sputum densities rebounded; the reduction in sputum inflammatory markers on the contrary was still present over 2 years. Microbiota composition analysis showed that the decrease in the relative abundance of *P. aeruginosa* was usually associated with an expansion of the diversity in the bacterial community, with a relative increase of commensal bacteria such as *Streptococcus*, *Prevotella*, *Veillonella* and other taxa.

A reduction of *P. aeruginosa* and SA airway colonization, associated with decreased numbers of antibiotic courses and maintenance treatment prescriptions was confirmed in a real-world setting by Hubert et al. [92] who carried out a retrospective multicentric study collecting clinical data in the year before and in the 2 years after IVA treatment in CF patients with at least one G551D mutation. The possibility of “clearance” of *P. aeruginosa* chronic respiratory infection after IVA therapy was reported in a case series by Strang and colleagues [93]. In this report, the clinical effectiveness of IVA treatment over 24 months was assessed in four paediatric Hispanic patients with S549N/F508del genotype. In three of these patients the treatment induced the eradication of chronic infection, that was associated with a remarkable improvement in lung function and growth. These findings seem to suggest the possibility of more striking changes in airway infection status than previously reported in patients with the G551D genotype; however, the very small number of patients do not allow any conclusion, but certainly deserves further studies.

The effect of IVA therapy on several microbiological markers was assessed by Millar et al. [94] in 15 adult CF patients with at least one G551D mutation that were followed from two years pre-IVA treatment to two years after commencement of treatment. A significant reduction in both the rate of isolation and the density of mucoid-*P. aeruginosa* was observed in these patients associated with a decrease in the number of IV antibiotic courses; in contrast there was no significant variation in both the rate of isolation and the density of non-mucoid *P. aeruginosa* and in oral antibiotic courses. This study did not report any reduction in antibiotic susceptibility of *P. aeruginosa* isolates following IVA treatment. A subsequent in vitro analysis from the same group confirmed that in vitro susceptibility of commonly used anti-pseudomonal antibiotics was not negatively affected by IVA [95].

One of the first studies investigating changes in airway microbiology associated with long-term IVA use was that of Frost et al. [96]. In this retrospective cohort study, data extrapolated from the UK CF Registry between 2011 and 2016 were analysed to determine the difference of annual prevalence ratios for key CF pathogens between IVA users and CF patients not under treatment with this modulator in the same time period. IVA use resulted in early and sustained reductions of positive sputum cultures for *P. aeruginosa*. In particular, the likelihood of a positive culture for this pathogen resulted in a reduction of 32% in this cohort after 3 years of treatment. Interestingly, the decrease in *P. aeruginosa* rates was the result of a combination of increased clearance in those already infected and reduced acquisition in those without infection.

Consistent with these results are the findings of a subsequent long-term large observational study assessing CF disease progression in patients treated with IVA in a real-world setting for up to 5 years [97]. This study used data from US and UK CF patient Registries to assess longitudinal changes in several clinical outcomes, including pulmonary exacerbations, hospitalizations, and *P. aeruginosa* prevalence in IVA-treated cohorts in comparison to controls patients matched by age, sex, and disease severity. During the 5 years follow-up CF patients treated with IVA had significantly lower frequencies of exacerbations and hospitalizations in comparison to pre-treatment baseline and comparators. Moreover, a favourable trend in *P. aeruginosa* prevalence was also observed.

Results of this study are supported by a recent multicentre, prospective, longitudinal, study [98] of CF patients aged ≥6 years with at least one copy of the G551D CFTR mutation followed up for 5.5 years. In these patients, significant reductions in *P. aeruginosa* detection and pulmonary exacerbation requiring antimicrobial therapy were observed, that were maintained through 5 years of treatment.

Harris et al. [99] compared sputum microbiome and markers of inflammation in CF patients with a G551D CFTR mutation before and after 6 months of IVA treatment and did not observe any significant change in airway microbial communities or measures of airway inflammation in the overall study cohort. Interestingly, younger patients and those without *P. aeruginosa* infection, and thus with less established lung disease, tended to experience more pronounced shifts in their microbial communities compared with older patients and *P. aeruginosa*–infected patient.

Very recently Einarrson et al. [71] used both extended-culture and culture-independent molecular methods to examine the effect of IVA on sputum bacterial communities in CF patients with G551 mutation. Differently from the previous study, extended culture and molecular culture-independent analysis demonstrated greater bacterial community richness and a trend towards greater diversity after IVA treatment. This shift towards a “healthier” lung microbiome was associated with reduced systemic inflammation and improvement in lung function in the subsequent year, suggesting that an increase in bacterial diversity richness may be linked to improvements in clinical status mediated by a downregulation of host inflammatory response. In this study, however, IVA treatment was not associated with a significant decrease in the amount of *P. aeruginosa* or total bacterial density.

Peleg et al. [100] evaluated the short-term effects of IVA on respiratory pathogens in patient with at least one G551D mutation and their relationship with concomitant antibiotic exposure in a double-blind, placebo-controlled study of one-month IVA treatment. No significant changes in the composition of CF airway microbiota have been observed during treatment with IVA compared to placebo, while any change in antibiotic exposure was associated with a significant change in microbiota composition, regardless of whether IVA or placebo was administered. Interestingly, in a small subgroup of subjects whose antibiotic exposure did not change during the study period, a significant reduction in total bacterial load was observed after initiation of IVA treatment.

At the 2021 North American Cystic Fibrosis Conference (NACFC), Singh et al. reported the results of a retrospective longitudinal cohort study analysing data from patients enrolled in the North American Cystic Fibrosis Foundation Patient Registry (CFFPR) between 2010 and 2017 [101]. This study evaluated the effect of CFTR modulators IVA and LUMA/IVA on new bacterial acquisition by longitudinal sputum culture analysis. In the group of patients treated with IVA, a decreased probability of acquiring a new infection with both SA and *P. aeruginosa* was reported (hazard ratio of 0.83, suggesting a 17% lower likelihood of acquiring a new infection), whereas a similar effect was not observed in the LUMA/IVA treated patients.

Finally, it is worth mentioning a retrospective monocentric study on a very small group of patients (*n* = 6) affected by ABPA in which clearance of *Aspergillus fumigatus* from sputum was observed after treatment with IVA (2 patients) or ETI (4 patients), even in patients with chronic *Aspergillus* infection over a ten-year period [102].

Patients with CF develop chronic infections mainly as a consequence of CFTR dysfunction leading to decreased mucociliary clearance, impaired local host defences and the inability to effectively clear bacteria from the airways. IVA has been shown to be able restore CFTR function and thus improve the altered airway microenvironment and induce remarkable effects in several clinical outcomes. Several studies have shown that this drug might reduce the bacterial burden in the airways and hinder the airway infections, and these effects may be the consequence of IVA-mediated increases in mucociliary clearance, local host defences or other mechanisms. The results of the different studies are however not always consistent; thus, further studies are needed on a larger number of patients with different gating mutations, to improve our knowledge of the long-term effectiveness and safety of IVA, and in particular to better understand the long-term microbiological consequences in treated CF patients and to predict future morbidity.

#### 4.1.2. Lumacaftor/Ivacaftor

On the basis of the evidence showing a synergistic effect of the association between correctors and potentiators, the second modulator introduced in clinical practice has been the association of LUMA (a CFTR corrector) and IVA (a CFTR potentiator), and subsequently of the corrector TEZA with IVA. A limited number of studies have assessed the effect of treatment with LUMA/IVA (LUMA/IVA) or TEZA/IVA (TEZA/IVA) on airway microbial communities changes. One of the most recent studies investigated the effects of LUMA/IVA on microbial composition and microbial metabolic activity in adult CF patients homozygous for the F508del mutation, in which repeated sampling of the lower respiratory tract was performed [77]. The overall results of this study did not show important effects of LUMA/IVA treatment on microbiological composition; in fact, only temporary and moderate changes in the lung microbiome were observed, mainly characterised by a reduction in the relative abundance of *P. aeruginosa*.

Different results were reported by Graeber et al. [76] in a prospective observational study of 30 CF patients (aged 12 years and older) with a F508del homozygous genotype treated for 8–16 weeks with LUMA/IVA. In this study, the treatment decreased the total sputum bacterial load (*p* < 0.05) and increased the Shannon diversity of the airway microbiome (*p* < 0.05), and these changes were associated with reduced interleukin-1β concentration in sputum.

Respiratory viral infections represent a significant cause of infectious exacerbations and hospitalization in CF patients, and in vitro findings suggest that dysfunctional CFTR reduces viral control by CF epithelial cells. To investigate whether treatment with modulators such as IVA or IVA/LUMA would improve the CF airways control of viral infection, De Jong et al. [103] assessed the in vitro response of CF airways epithelial cells to rhinovirus after exposure to LUMA/IVA. They showed that CFTR modulation does not have a significant impact on the CF epithelium response to virus, although it may affect some of the metabolic and inflammatory aspects known to be dysregulated in the CF airway epithelium.

#### 4.1.3. Elexacaftor/Tezacaftor/Ivacaftor

Recently, the three-drug combination ETI was approved for individuals with at least one F508del allele; as F508del is the most common CF mutation worldwide, ETI has the potential to treat the large majority of CF patients. Randomized controlled trials of ETI demonstrated significant improvements in lung function, respiratory symptoms, weight gain and risk of acute pulmonary exacerbations. The approval of ETI thus represented a milestone advance in managing CF.

Thus far, few data are available about the effects of ETI on chronic airway infections as well as on CF bacterial community. In this context, one of the most recent studies is that from Sosinski et al. [78] that we previously mentioned. They analysed sputum microbiome and metabolome from CF patients before and after ETI therapy and showed that the treatment was associated with a change of microbiome and metabolome in the airways. These findings seem to suggest that treatment with ETI might “reshape microbiome niche space in CF mucus”, an effect potentially relevant for treatment of airway infections in CF.

Sheikh et al. [104] evaluated the impact of 6 months treatment with ETI on clinical parameters, including microbiological cultures and inflammatory/immune mediators in 32 CF patients. A significant decrease of culture positivity for *P. aeruginosa* was observed after treatment as compared to basal condition (32% vs. 55% respectively, *p* < 0.02) that was associated with a significant improvement of FEV1 and BMI and decreased sweat chloride levels; an improvement in blood inflammatory markers including the cytokines IL-8 and IL-17A, was also reported.

In a retrospective analysis of data collected from a large number of CF patients (250) treated with ETI, sputum cultures were analysed (both per patient and per total culture) for the presence or absence of pathogens 1 year before and 1 year after starting ETI. A statistically significant reduction in colonization by all pathogens was observed after starting ETI both in patients with and without previous exposure to other modulators (the relative reduction rate was: 34% for *P. aeruginosa*, 21% for MSSA, 25% for MRSA, 54% for *Achromobacter*, 68% for *S*M and 60% for *B. cepacia*) [105].

At the 2021 NACFC, Morgan et al. presented preliminary results on the effect of ETI on the sputum microbiome of 51 CF patients enrolled in the observational study “PROMISE” [106]. In this study, induced sputum was collected for microbiome analysis at 1, 3, 6 and 12 months after initiating ETI. This preliminary report shows that treatment with ETI is capable of inducing a rapid decrease in the relative abundance of predominant CF pathogens (average relative abundance declined by 15% for *P. aeruginosa* after 1 month and by 28% for *S. aureus*) with a concomitant increase in the relative abundance of other organisms such as *Streptococcus*, *Prevotella* and *Veilonnella*.

Changes in the microbiome profiles after treatment with ETI, primarily driven by an increase in the relative abundance of anaerobes, particularly *Veilonnella* spp., were confirmed by Quinn et al., who did not observe, however, changes in relative abundance of *P. aeruginosa* [107].

Thus, although the introduction of ETI in the clinical arena is very recent, it seems well established that the majority of treated patients will obtain relevant benefit from ETI therapy; however, as variability is expected in both effects on CFTR function and clinical status, long-term studies will allow to better define the mechanisms and the clinical impact of this therapy, and in particular its role in modulating CF airway infections.

In Table 2 are summarized the most recent studies addressing the effects of CFTR modulators on airway microbiology/infections.

### 4.2. CFTR Modulators and Recurrent Infectious Exacerbations

#### 4.2.1. Ivacaftor

As we already mentioned, the first modulator introduced in clinical practice for patients with CF aged ≥12 years with a G551D CFTR mutation was IVA. The first studies [80,108] showed that IVA induced a 55% reduction of pulmonary exacerbations at 48 weeks as compared to placebo. Furthermore, it also reduced the duration of exacerbations, as well as the number of days of hospitalization. As already mentioned, in the following years IVA was granted a series of label expansions and is now approved for patients as young as 4 months-old and in patients with several gating mutations other than G551D.

Duckers et al. [75] in a systematic review on the effects of IVA confirmed that this potentiator significantly and consistently reduces the number of pulmonary exacerbations. A subsequent study by Salvatore and colleagues focused on the effect of IVA in patients with severe lung disease; in particular, they studied patients with an FEV1 <40% predicted in the previous 6 months or being on a lung transplant waiting list and/or exhibiting a severely worsening trend of lung function (FEV1 loss > 10% during the previous year) and showed a significant reduction of pulmonary exacerbations after 12 months of treatment with IVA [109].

Among the real-life studies on modulators focusing on IVA, that of Fink and colleagues conducted on data from US Registry showed an annual decrease of 1.2 exacerbations after 12 months of treatment in patients of 6 years of age and older [110]. In an observational study using data from the US and UK Cystic Fibrosis Registries, Bessonova et al. observed a significant reduction of pulmonary exacerbations in patients treated with IVA for 2 or 3 years after commercial drug availability as compared with a control population (28.7% vs. 43%); interestingly, the observed reduction of exacerbations was similar throughout all age groups and the different pulmonary function values [111]. Consistent with these results, Hubert et al. in a retrospective multicentre observational study, reported a significant reduction in both oral and IV antibiotics courses, as well as in their duration [92].

As already emphasized, up to now the literature concerning long-term effects of modulators including IVA is quite limited and only few studies have been published. Interestingly, Volkova et al., in one of the biggest real-life studies on CFTR modulators conducted using US (635 patients) and UK Registries (247 patients), assessed the effects of IVA during a 5-year observation period and confirmed a significant reduction of pulmonary exacerbations in treated patients that persisted throughout the overall observation period [97]. An additional real-world study is the already quoted study by Guimbellot et al. [98] that assessed the effects of IVA after 5.5 years of treatment in CF patients ≥ 6 years old, with at least one copy of G551D CFTR mutations (*n* = 96). In this study, the reduction of pulmonary exacerbations was persistent during the study time: whereas 36.2% of patients experienced a pulmonary exacerbation during the year prior the starting of IVA, in only 16% an exacerbation occurred during the 0.5–1.5 year-time interval after the beginning of treatment and in 16.7% during the 4.5–5.5 year-time interval. Another longitudinal study assessed the effect of IVA treatment in 114 patients from Canadian Registry, with either G551D and non-G551D mutations; 50% of these patients were treated with IVA for more than 4 years and some for up to 9 years. The authors observed [112] an 18% decrease in pulmonary exacerbations after IVA treatment, although the reduction was not statistically significant, possibly due to the inclusion of individuals with milder clinical phenotypes.

#### 4.2.2. Lumacaftor/Ivacaftor

The combination of LUMA and IVA has been available since 2015. In the US, FDA approval was based on two 24-week trials of 1108 patients ≥ 12 years, homozygous for F508del mutation (TRAFFIC and TRANSPORT) showing that this association was capable of significantly reducing pulmonary exacerbations by 30–39% in treated patients (*p* < 0.001) and inducing a decrease of events leading to hospitalization and the use of IV antibiotics [113]. Subsequent subgroup analyses demonstrated that LUMA/IVA therapy was associated with a reduced exacerbations rate regardless of patient baseline characteristics including FEV1, age, sex, medication use, and *P. aeruginosa* status [114].

As with IVA, the association LUMA/IVA was granted a number of label extensions [115,116] and it is now approved for patients with CF ≥ 2 years old, homozygous for F508del mutation. A subsequent extension of the TRAFFIC and TRASPORT studies (PROGRESS study) [117] assessing the effects of LUMA/IVA after 96 weeks of treatment, confirmed the persistent reduction of pulmonary exacerbations rate observed in the TRAFFIC and TRANSPORT studies, as well as the decrease of severe events leading to hospitalization and the use of IV antibiotics.

Two subsequent studies assessed the response to LUMA/IVA in patients with CF ≥ 12 years old, homozygous for F508del, with severe pulmonary disease (FEV1 < 40%). The first study included 46 patients treated for 24 weeks [118] (28 of them with full drug dose and 18 administered half-dose). This study showed a significant reduction in IV-antibiotics course duration (M [SD]: −8.52 [24,91] days, *p* = 0.03) and in all-causes of hospitalization rate. The second study was a multicentre case control study [119] performed on 72 patients treated with LUMA/IVA for 12 months and on 33 control subjects matched for age, sex, and lung function. In this study, a reduction in pulmonary exacerbations was observed associated with an increase in the time to first exacerbation. Burgel et al. in a real-life study confirmed that LUMA/IVA resulted in a reduction of intravenous antibiotics courses in adolescents and adults homozygous for F508del who tolerated the treatment; however, they also showed that the risk of treatment discontinuation due to adverse events was increased in patients with low lung function or repeated exacerbations, and in adults versus adolescents. Overall, these findings support the role of LUMA/IVA in decreasing the number of pulmonary exacerbations. However, further data are still needed to confirm this effect in patients with severe pulmonary disease [120,121]. We also need further data concerning long-term effects of this drug on pulmonary exacerbations.

#### 4.2.3. Tezacaftor/Ivacaftor

In 2018, the association of TEZA and IVA was approved. Comparably to the LUMA/IVA association, also in this case the two approval studies [122,123] showed a significant reduction of pulmonary exacerbations rate that was 35% lower in the treated group as compared to placebo. Again, it is important to emphasize that further data would be required to confirm these effects, in particular in specific subsets of patients, and in the long-term.

#### 4.2.4. Elexacaftor/Tezacaftor/Ivacaftor

As we already highlighted, the discovery and introduction in clinical practice of ETI, an association of two correctors with different mechanism of action and a potentiator set the stage for an impressive improvement in CF disease outcome. Concerning the effects on pulmonary exacerbations, a phase 3, randomised, double-blind, placebo-controlled trial conducted in patients with CF ≥ 12 years old, heterozygous for the F508del CFTR mutation and a minimal-function mutation, has shown that the treatment with ETI resulted in a 63% lower annualized rate of exacerbations compared to placebo [64]. This and another earlier study [65] provided evidence of a clear-cut superiority of ETI compared to TEZA/IVA. Further studies are required to determine long-term effects of ETI, as well as real-life effectiveness and possible implications of beginning the treatment during the early stages of disease.

A recent study performed on a small cohort (14 patients) with advanced lung disease (FEV1 < 40% and/or being on an active lung transplantation list) reported a reduction in infective exacerbations requiring hospitalization during a follow-up period of 4.9 months [124].

The interim analysis from a recent and still ongoing US-based multicentre observational study of 16 months duration (the HELIO study [125]) in patients treated with ETI showed that the annualized pulmonary exacerbations rate was significantly lower during the treatment period (6 months) than in the year before the drug initiation.

A very recent study on patients enrolled in the CF Foundation Patient Registry at the Columbia University adult CF program focused on the evaluation of pulmonary exacerbations rate during the years 2019 and 2020 in patients on ETI as compared to those not on ETI [126]. Although a reduction in exacerbations was expected and observed as a consequence of the restrictive measures adopted for COVID-19 pandemic; however, in the group of patients on ETI a significantly greater reduction of exacerbations was registered as compared to the control group. This effect was observed for both mild/moderate (those resulting in oral antibiotic prescriptions) and severe exacerbations (those requiring IV antibiotics or hospitalization) with a decrease of 76% and 88.5%, respectively, in the ETI group as opposed to 26% and 36.4% in the control group. In Table 3, we have summarized the most recent studies addressing the effects of CFTR modulators on pulmonary exacerbations.

In the last decade, the introduction of CFTR modulator therapy has radically changed the course of CF. However, we still need further evidence on its role in pulmonary exacerbations in various subgroups of patients and in the long-term.

## 5. Conclusions

Over the last decade, the survival of patients with CF has increased considerably as a result of increased knowledge on disease mechanisms. The consequent improvements in clinical care, in particular, the recent introduction in the clinical arena of CFTR modulator therapy has had an impressive impact on the health outcomes and quality of life of CF patients. By restoring or increasing CFTR function, modulator drugs correct the pathophysiological mechanisms underlying CF disease.

However, despite the impressive improvements in CF care and patients’ survival, microbial infections of the airways remain a significant clinical problem in CF. Chronic airway infections and recurrent acute exacerbations are in fact responsible for most of the morbidity and mortality in people with CF and largely contribute to progressive tissue damage and lung function impairment. Thus, therapeutic strategies aimed at preventing and controlling airway infections remain a major goal of CF treatment.

The management of bacterial infection in CF lungs has benefited from advanced antibiotic regimens associated with mucolytics and regular physical therapy. The microbiology of the CF lung is also changing, as the novel therapeutic strategies have reduced the ability of bacteria to colonise into the airway mucus and the airway epithelium. However, as we previously highlighted, although progress is being made against all major CF pathogens, problems of persistence and reinfection continue to exist. CF pathogens have different prevalence within age groups, and during the disease course; furthermore, the microorganisms may have developed specific resistance mechanisms, enhancing their persistence into the CF airways. In addition, there is now growing evidence that interactions among members of the microbial community play an important role in clinical outcomes as well as in lung disease progression and the response to therapy.

The introduction to clinical practice of successful CFTR modulators therapy has shown remarkable effects on clinical outcome in CF patients and is also regarded as a potential strategy that may positively affect lung infections.

To date, CFTR modulator studies have focused largely on physiological and clinical effects, including lung function, symptoms, sweat chloride levels and disease exacerbations; the evaluation of changes in airway microbiology and the effects on development and/or persistence of chronic airway infections were not addressed in depth. Thus, so far little is known about the full extent of CFTR modulator’s effects on CF pathogens as well as on the long-term effects on chronic airway infections and infectious exacerbations, and some inconsistent results have been reported.

Several studies are now ongoing, addressing the crucial role of novel modulator therapies in decreasing the long-term incidence/prevalence of chronic and recurrent airway infections; taken together, the preliminary results of these studies suggest that the available CFTR modulators, particularly IVA, may favourably impact CF microbiology, and are capable of decreasing the prevalence of bacteria particularly the key CF pathogen *P. aeruginosa* in treated patients, although contrasting results have been reported and the long-term duration of these effects is currently unclear.

Moreover, some in vitro studies have shown direct antimicrobial properties of certain modulators and a synergistic interaction between CFTR modulators and certain antibiotics. However, also in this case, further studies are needed to confirm and extend upon these findings.

In conclusion, although CFTR modulators have shown the capability of decreasing disease exacerbations and airway bacterial burden in CF patients, the long-term effects either on chronic infections or on acute exacerbations are yet to be clearly defined and many questions remain to be answered. Understanding the impact of CFTR modulators on CF airway microbiology and the effects of these new drugs on chronic and acute airway infections, as well as evaluating their effects in combination with antibiotics, will extend our current understanding of the mechanisms of airway infection and will play an essential role in developing and improving strategies for managing airway infections in CF.

Highly effective CFTR modulator therapy is now available to an increasing proportion of CF patients and has led to impressive improvements in CF care and patient survival. Thus, it is expected that this therapy will be increasingly used early in the disease course before the development of bacterial infection, thus with the high potential to prevent the occurrence of airway infection. However, effective CFTR modulator therapies are not yet available for patients with rarer mutations. Furthermore, there is also a variability in patient response to existing modulators. Thus, antimicrobial therapy and all traditional strategies aimed at preventing or controlling airway infections still maintain a crucial role in CF treatment.

## Figures and Tables

**Table 1:** Approved CFTR modulators.

Modulator (Commercial Name)	Responsive Mutations	Age Eligibility	Approval Year
Ivacaftor (Kalydeco^®^ USA/EU)	G551D, S549N, G1244E, G178R, S1251N, G551S, G1349D, S1255P, R117H, E56K, K1060T, P67L, E193K, A1067T, R74W, L206W, G1069R, D110E, R347H, D579G, R1070Q, D1270N, D110H, R352Q, S945L, R1070W, R117C, A455E, S977F, F1074L, F1052V, D115H; 3849 + 10 kb C>T, 2789 + 5G>A, 3273-26A>G, 711 + 3A>G, E831X	≥4 months	2012
Lumacaftor-Ivacaftor (Orkambi^®^ USA/EU)	Two copies of F508del	≥2 years	2015
Tezacaftor-Ivacaftor (Symdeko^®^ USA) (Symkevi^®^ EU)	Two copies of F508del One copy of F508del in association with E56K, K1060T, P67L, E193K, A1067T, R74W, L206W, D110E, D110H, R347H, D579G, R1070Q, D1270N, R352Q, S945L, R1070W, R117C, A455E, S977F, F1074L, F1052V, D1152H, 3849 + 10 kb C>T, 2789 + 5G>A, 327326A>G, 711 + 3A>G	≥6 years	2018
Elexacaftor-Tezacaftor-Ivacaftor (Trikafta^®^ USA) (Kaftrio^®^ EU)	One copy of F508del	≥12 years	2019 (USA) 2020 (EU)

**Table 2:** Effects of CFTR modulators on airway microbiology/infections.

Study			Patients’ Characteristics			Findings (Related to Airway Microbiology/Infection)
First Author; Year [Reference]	CFTR Modulator ^1^	Treatment Duration	Patients Number	Age (Years)	Genotype ^2^	Airway Microbiology/Infections ^3^
Rowe S.M.; 2014 [90]	IVA	6 months	133	≥6	G551D	↓ PA burden and ↑ Prevotella
Heltshe S.L.; 2015 [91]	IVA	6 months	151	≥6	G551D	29% of patients positive for PA the year prior to IVA use were culture negative the year following treatment 88% of those PA free remained uninfected. No change in SA or MRSA
Hisert K.B.; 2017 [73]	IVA	2 years	12	≥18	G551D	↓ PA load but any eradication ↑ Streptococcus, Prevotella, Veilonnella
Hubert D.; 2018 [92]	IVA	2 years	57	≥6	G551D	↓ PA and SA colonization ↓ N. of antibiotic courses
Strang A.; 2017 [93]	IVA	2 years	4	10–16	S549N/F508del	Eradication of PA in 3 patients
Millar B.C.; 2018 [95]	IVA	2 years	15	≥18	G551D	↓ Rate of isolation of mucoid-PA ↓ Density of M-PA
Frost F.L.; 2019 [96]	IVA	5 years	276 vs. 5296	≥6	G551D	↓ PA in sputum
Volkova N.; 2020 [97]	IVA	5 years	635 vs. 1874 comparators	0–≥18	Class I-III	↓ PA prevalence
Guimbellot J.S.; 2021 [98]	IVA	5.5 years	96	≥6	G551D	↓ PA prevalence
Harris J.K.; 2020 [99]	IVA	6 months	31	≥10	G551D	Any significant change
Einarsson G.C.; 2021 [71]	IVA	1 year	14	≥13	G551D	Greater bacterial diversity, “healthier” microbiome. No change in PA infection
Peleg A.Y.; 2018 [100]	IVA	1 month	20	≥18	G551D	Not significant change in microbiota in IVA vs. placebo. Significant changes associated with any change in ATB exposure
Singh S.; 2021 [101]	IVA LUMA/IVA	3 years	173	≥12	G551D F508del/F508del	Only in IVA group ↓ new infection with both SA and PA.
Neerincx A.H.; 2021 [77]	LUMA/IVA	1 year	20	≥18	F508del/F508del	No significant effect on microbiological composition
Graeber S.Y.; 2021 [76]	LUMA/IVA	8–16 weeks	30	≥12	F508del/F508del	↓ Total sputum bacteria load ↑ Diversity in airway microbiome
De Jong E.; 2021 [103]	LUMA/IVA	NA ^4^	In vitro study	NA	F508del/F508del F508del/G551D	No significant impact of LUMA/IVA on CF epithelium response to Rhinovirus
Sosinski LM; 2021 [78]	ETI	1 year	24	≥18	At least one copy of F508del	Reshape microbiome niche space in CF mucus

^1^: IVA: Ivacaftor, LUMA: Lumacaftor, ETI: Elexacaftor/Tezacaftor/Ivacaftor. ^2^: MF: minimal function. ^3^: ↓ = reduction, ↑ = improvement/increase, PA: *Pseudomonas aeruginosa*, SA: *Staphylococcus aureus*, MRSA: Methicillin-Resistant *Staphylococcus aureus*, M-PA: mucoid *Pseudomonas aeruginosa*, ATB: antibiotics, IV: intravenous. ^4^: NA: not applicable.

**Table 3:** Effects of CFTR modulators on pulmonary exacerbations.

Study			Patients’ Characteristics			Findings (Related to Pulmonary Exacerbations)
First Author; Year [Reference]	CFTR Modulator ^1^	Treatment Duration	Patients Number	Age (Years)	Genotype ^2^	Pulmonary Exacerbation ^3^
Duckers J.; 2021 [75]	IVA	Between 2012–2019	≥6 for each study	≥12	G551D	↓
Ramsey B.; 2011 [80]	IVA	48 weeks	84 IVA/83 placebo	≥12	G551D	↓ 55% vs. placebo
Salvatore D.; 2019 [109]	IVA	12 months	13 with severe lung disease	≥10	At least one CFTR gating mutation (G178R, S549N, S549R, G551S, G970R, G1244E, S1251N, S1255P, and G1349D)	↓ Mean number of PEx/patient/year from 4.38 (1.8) before to 2.15 (1.99) after starting IVA
Fink A.; 2015 [110]	IVA	1 year	403	≥6	G551D	Mean difference −1.2 (SD:1.1)
Bessonova L; 2018 [111]	IVA	2 years	1667 IVA vs. 8269 comparators	0–≥18	Class I-III Class IV-VI Unknown	27.8% IVA vs. 43.3% comparators
Kawala C.R.; 2021 [112]	IVA	>4 up to 9 years	144	Median (IQR): 22.5 (11.1–34.4)	Class III (124) Class IV–V (20)	↓ 18% (non-significant)
Wainwright C.E.; 2015 [113]	LUMA/IVA	24 weeks	1108	≥12	F508del/F508del	↓ 30–39% vs. placebo
McColley S.A.; 2019 [114]	LUMA/IVA	24 weeks	369	≥12	F508del/F508del	↓ even in patients without early lung function improvement.
Konstan WN; 2017 [117]	LUMA/IVA	96 weeks	1030	≥12	F508del/F508del	↓ compared to placebo
Taylor-Cousar J.L.; 2018 [118]	LUMA/IVA	24 weeks	46	≥12	F508del/F508del	↓ Annualized hospitalization rate (rate ratio: 0.41) ↓ IV atb duration (mean difference: −8.52 days) through study week 24.
Tong K.; 2020 [119]	LUMA/IVA	12 months	72	≥12	F508del/F508del	↓ ↑ Time to first exacerbation
Taylor-Cousar J.L.; 2017 [122]	TEZA/IVA	24 weeks	510	≥12	F508del/F508del	↓ 35% in the TEZA/IVA group than in the placebo group
Rowe S.M.; 2017 [123]	TEZA/IVA	8–16 weeks	248	≥12	F508del/F508del	↓ Rate but not statistically significant
Middleton P.G.; 2019 [64]	ETI	24 weeks	403	≥12	F508del/MF	↓ 63% vs. placebo
O’Shea K.M.; 2021 [124]	ETI	4.9 months	14 (severe lung disease)	19–46	F508del/F508del F508del/MF	↓ Exacerbations requiring hospitalization
Ganapathy V; 2021 [125]	ETI	96 weeks	100	≥12	F508del/MF F508del/F508del	↓ from 1.24 to 0.9

^1^: IVA: Ivacaftor, LUMA: Lumacaftor, TEZA: Tezacaftor, ETI: Elexacaftor/Tezacaftor/Ivacaftor. ^2^: MF: minimal function. ^3^: ↓ = reduction, ↑ = improvement/increase, PEx: pulmonary exacerbation, ATB = antibiotics, IV = intravenous.

## Data Availability

Not applicable.
